# Comparison of ^99m^Tc-3PRGD2 Integrin Receptor Imaging with ^99m^Tc-MDP Bone Scan in Diagnosis of Bone Metastasis in Patients with Lung Cancer: A Multicenter Study

**DOI:** 10.1371/journal.pone.0111221

**Published:** 2014-10-22

**Authors:** Weibing Miao, Shan Zheng, Haojie Dai, Feng Wang, Xiaona Jin, Zhaohui Zhu, Bing Jia

**Affiliations:** 1 Department of Nuclear Medicine, the 1st Affiliated Hospital of Fujian Medical University, Fuzhou, China; 2 Department of Nuclear Medicine, Beijing Tongren Hospital, Beijing, China; 3 Department of Nuclear Medicine, the 1st Affiliated Hospital of Nanjing Medical University, Nanjing, China; 4 Department of Nuclear Medicine, Peking Union Medical College Hospital, Chinese Academy of Medical Sciences and Peking Union Medical College, Beijing, China; 5 Medical Isotopes Research Center, Peking University, Beijing, China; University of L'Aquila, Italy

## Abstract

**Purpose:**

^99m^Tc-3PRGD2, a promising tracer targeting integrin receptor, may serve as a novel tumor-specific agent for single photon emission computed tomography (SPECT). A multi-center study was prospectively designed to evaluate the diagnostic accuracy of ^99m^Tc-3PRGD2 imaging for bone metastasis in patients with lung cancer in comparison with the conventional ^99m^Tc-MDP bone scan.

**Methods:**

The patients underwent whole-body scan and chest tomography successively at both 1 h and 4 h after intravenous injection of 11.1 MBq/Kg ^99m^Tc-3PRGD2. ^99m^Tc-MDP whole-body bone scan was routinely performed within 1 week for comparison. Three experienced nuclear medicine physicians blindly read the ^99m^Tc-3PRGD2 and ^99m^Tc-MDP images. The final diagnosis was established based on the comprehensive assessment of all available data.

**Results:**

A total of 44 patients (29 male, 59±10 years old) with suspected lung cancer were recruited from 4 centers. Eighty-nine bone lesions in 18 patients were diagnosed as metastases and 23 bone lesions in 9 patients were benign. In a lesion-based analysis, ^99m^Tc-3PRGD2 imaging demonstrated a sensitivity, specificity, and accuracy of 92.1%, 91.3%, and 92.0%, respectively. The corresponding diagnostic values for ^99m^Tc-MDP bone scan were 87.6%, 60.9%, and 82.1%, respectively in the same patients. ^99m^Tc-MDP bone scan had better contrast in most lesions, whereas the ^99m^Tc-3PRGD2 imaging seemed to be more effective to exclude pseudo-positive lesions and detect bone metastases without osteogenesis.

**Conclusion:**

^99m^Tc-3PRGD2 is a novel tumor-specific agent based on SPECT technology with a promising value in diagnosis of bone metastasis in patients with lung cancer.

**Trial Registration:**

ClinicalTrials.gov NCT01737112

## Introduction

Lung cancer is one of the most fatal diseases worldwide. Early diagnosis and accurate evaluation of bone metastasis of lung cancer are essential for therapeutic planning and relapse monitoring [1]–[3]. Bone scan using ^99m^Tc-MDP as the tracer is applied to detect bone metastasis. However, its diagnostic value is limited because of the low specificity.

Integrin is mainly involved in the cell-cell and cell-matrix interactions. Integrin α_v_β_3_, an important member of the integrin family, is highly relevant to neoplastic angiogenesis, invasion, and metastasis. It is not expressed at all or expressed at a very low level on the quiescent endothelium and other normal tissues [4]–[8]. Therefore, the integrin α_v_β_3_ receptor may be a promising target in the diagnosis, evaluation, and treatment of malignancies [9]–[12], serving as a tumor-specific agent for single photon emission computed tomography (SPECT).

The tri-peptide sequence of arginine-glycine-aspartic acid (RGD), especially in the cyclic form, has shown high affinity in binding with the receptors of integrin α_v_β_3_. Some radiolabeled cyclic RGD monomer peptides, such as ^18^F-Galacto-RGD, ^18^F-AH111585, and ^99m^Tc-NC100692, have thus been translated into clinical use and showed potential in the detection of breast and lung cancers [13]–[20]. ^99m^Tc-HYNIC-3PEG_4_-E[c(RGDfK)_2_] (^99m^Tc-3PRGD2 in short) is a new cyclic RGD dimmer peptide separated by three PEGs. It shows much higher affinity to integrin α_v_β_3_, which enables its higher tumor accumulation than the prior RGD monomer tracers [21]–[27]. As a SPECT tracer, it is cost-effective and broadly available. Most importantly, it may serve as a novel SPECT tracer specific for the detection of malignant tumors.

In a prior study, we have preliminarily demonstrated that ^99m^Tc-3PRGD2 imaging at 1 h after injection was sensitive for the detection of lung cancer [28]. This prospective multi-center study was designed to investigate the value of ^99m^Tc-3PRGD2 as compared with ^99m^Tc-MDP in the assessment of bone metastasis of lung cancer.

## Materials and Methods

The protocol for this trial and supporting CONSORT checklist are available as supporting information; see Checklist S1 and Protocol S1.

### Patients

A total of 44 patients (29 males and 15 females; aged from 41 to 76 years old, mean age ±SD, 59.2±9.7) with suspected lung lesions were recruited from 4 centers in China from Feb. 2011 to Jan. 2014, and their definite pathological diagnoses were obtained (Table 1). This study had been approved by the Institute Review Board of Peking Union Medical College Hospital, Chinese Academy of Medical Sciences & Peking Union Medical College firstly and then by each center, including the 1^st^ Affiliated Hospital of Fujian Medical University, Beijing Tongren Hospital and the 1^st^ Affiliated Hospital of Nanjing Medical University. It was part of the clinical trial registered online at NIH ClinicalTrial.gov (NCT01737112). The main reason for the delay in registering the study was to protect the idea of the study. The authors confirm that all ongoing and related trials for this drug/intervention are registered. All the participants provided their written informed consent to participate in this study, and the records were saved by each center. All the patients were identified with suspected primary lung cancer in accordance with the findings of computed tomography (CT) performed within 2 weeks. The “standard of truth” for diagnosis of the lung lesions was based on histopathological findings of surgery or fine needle or bronchoscopic biopsy, and for that of bone lesions was based on the comprehensive analysis of the outcome. The diagnosis of all the bone lesions was confirmed from at least 2 imaging modalities (SPECT/CT/MRI/^18^F-FDG PET-CT) and 6-month follow-up (Table 2). Patients' clinical records were also taken into consideration. Consensus reading of ^99m^Tc-3PRGD2 and ^99m^Tc-MDP images was performed on the same workstation by three experienced nuclear medicine physicians.

**Table 1:** The recruited patient number and SPECT systems of the 4 centers.

Centers	Recruited patients	SPECT system
**The 1^st^ Affiliated Hospital of Fujian Medical University, Fuzhou**	**21**	**GE Infinia Hawkeye**
**Beijing Tongren Hospital, Beijing**	**11**	**GE Infinia Hawkeye4**
**The 1^st^ Affiliated Hospital of Nanjing Medical University, Nanjing**	**8**	**Siemens Symbia T6**
**Peking Union Medical College Hospital, Beijing (The organizing center)**	**4**	**Philips Precedence 16**

**Table 2:** Flowchart of the study.

RESEARCH PROCESS	V1	V2	V3	V4(Surgery)	V5(Follow-up)
Window period		Less than two weeks interval to V4	Less than one week interval to V2		once every 3–6 months
Inclusion/Exclusion criteria	X				
Signed Informed Consent Form	X				
Medical history/Concomitant diseases	X				
CT, MRI, PET/CT	X				
Tumor markers and other laboratory tests	X				
^99m^Tc-3PRGD_2_ SPECT		X			
^99m^Tc-MDP whole body scan			X		
Adverse events		X			
Surgical records and pathology results				X	
Preserved specimens				X	
Subsequent treatment and follow-up					X

### Radiopharmaceutical preparation

The kit for preparation of ^99m^Tc-3PRGD2 was supplied by the Medical Isotopes Research Center, Peking University. Synthesis of the labeling precursor, kit preparation, and ^99m^Tc-labeling were previously described [27]. ^99m^Tc-MDP was supplied by the Atom High-tech Company of China. The radiochemical purity was >95%.

### Imaging protocol

All the scanners were dual-head gamma-cameras, using low-energy high-resolution collimators and a 20% energy window centered on 140 keV. After intravenous injection of 11.1 MBq/kg ^99m^Tc-3PRGD2, whole body planar scan (10 cm/min) and chest SPECT imaging (zoom ×1, 30 sec/frame/6°) were performed at dual time-points of 1 h and 4 h (^99m^Tc have a half life of 6 h). ^99m^Tc-MDP whole body images were acquired at a scan speed of 10 cm/min within 1 week before or after ^99m^Tc-3PRGD2 imaging.

### Image and statistical analyses

Visual image analysis was completed through consensus reading by 3 experienced nuclear medicine physicians blind to the history and pathological diagnosis. All the bone lesions were evaluated lesion by lesion on both whole-body scan and thoracic SPECT images. The image quality, number of lesions, and diagnosis (benign or malignant) were documented.

The sensitivity, specificity, accuracy, positive predictive value (PPV), and negative predictive value (NPV) were employed to determine the efficacy of ^99m^Tc-3PRGD2 imaging for the diagnosis of bone metastases. The corresponding parameters of ^99m^Tc-MDP were also calculated for comparison. Chi square test (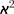 test) was used to make comparison of the rates of different samples, with *P*<0.05 considered significant by using SPSS 15.0 software package.

## Results

Among the 44 recruited patients, 38 were confirmed to have pulmonary malignancy, including adenocarcinoma (n = 18), squamous cell carcinoma (n = 13), small cell lung cancer (n = 3), adeno-squamous carcinoma (n = 1), metastatic adenocarcinoma (n = 2), and metastatic melanoma (n = 1); 6 were diagnosed with benign lesions, including inflammatory pseudo-tumor (n = 2), granuloma of tuberculosis (n = 2), and fungal infection (n = 2).

**Figure 1:**
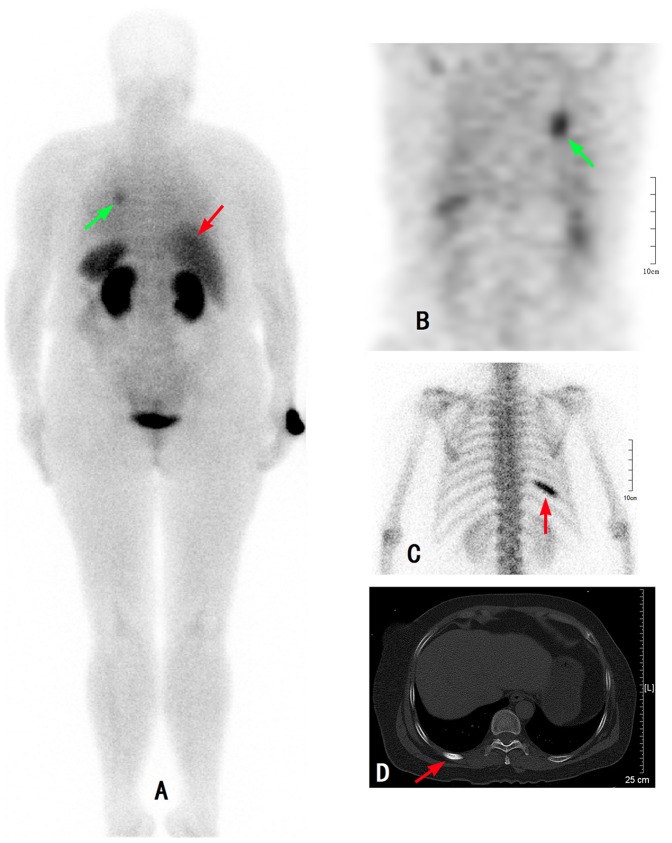
Comparison of ^99m^Tc-3PRGD2 SPECT, ^99m^Tc-MDP bone scan (posterior), and CT scan in a 62 year-old woman with left upper lung adenocarcinoma (green arrow) and costal metastasis (red arrow). A:^ 99m^Tc -3PRGD2 WB(+), B: ^99m^Tc -3PRGD2 SPECT (+), C: ^99m^Tc -MDP bone scan (+), D: CT (+). WB: whole body.

**Figure 2:**
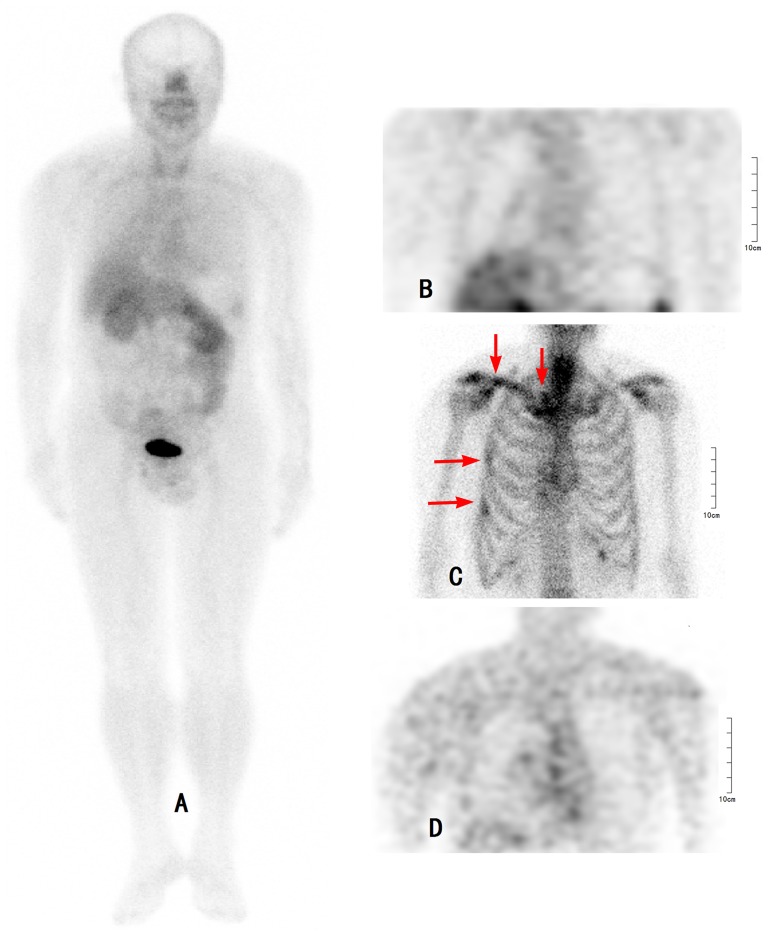
Comparison of ^99m^Tc-3PRGD2 SPECT, ^99m^Tc -MDP bone scan, and ^18^F-FDG PET in a 60 year-old patient with a recent fall-down trauma. The^99m^Tc -MDP bone scan was positive (red arrows), whereas both ^99m^Tc-3PRGD2 SPECT and ^18^F-FDG PET were negative. A: ^99m^Tc-3PRGD2 WB (-), B: ^99m^Tc-3PRGD2 SPECT (−), C: ^99m^Tc-MDP bone scan (+), D: ^18^F-FDG PET (−). WB: whole body.

**Figure 3:**
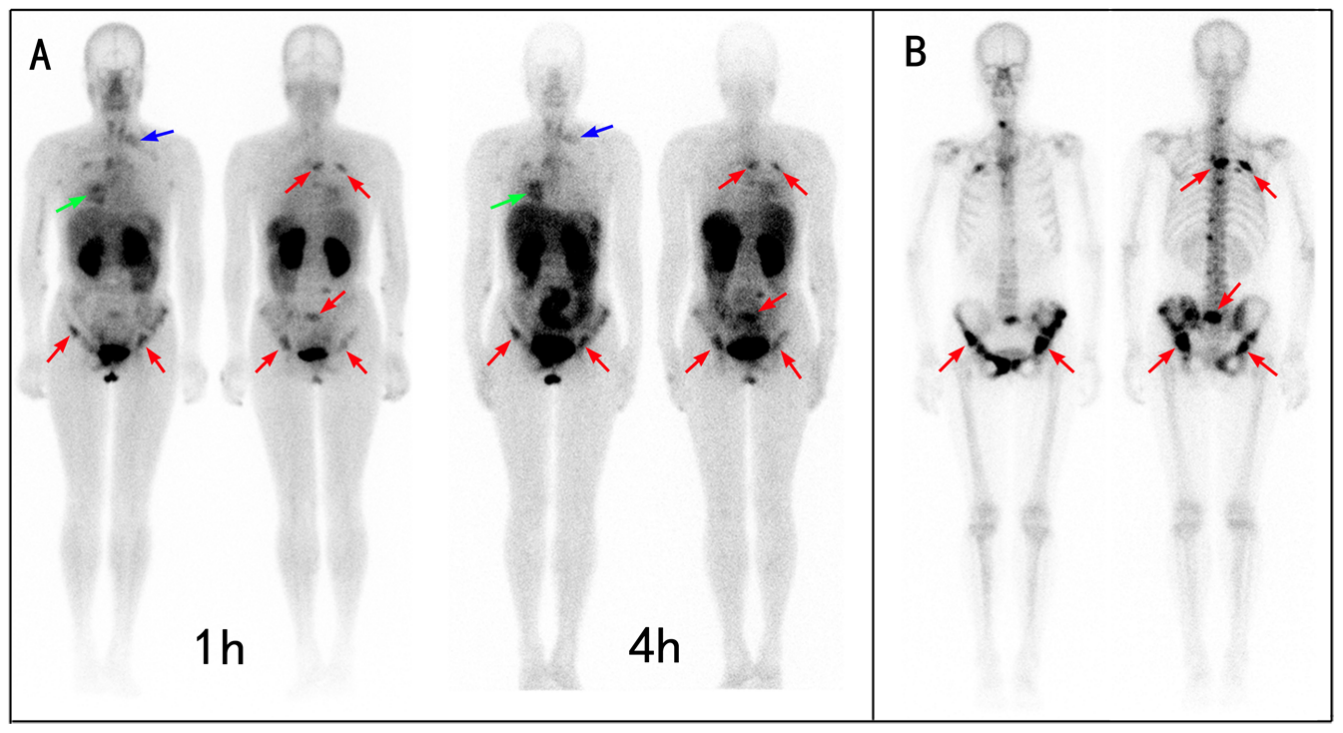
Comparison of ^99m^Tc-3PRGD2 integrin receptor imaging with ^99m^Tc-MDP bone scan in a patient with multiple bone metastases. A: The ^99m^Tc-3PRGD2 imaging showed the lung cancer (green arrow), lymph node metastases (blue arrow), and bone metastases (red arrow) at the same time. The 1-h imaging is better than the 4-h imaging because of the relatively lower background in bone marrow, liver, and spleen. B. ^99m^Tc-MDP bone scan demonstrated better contrast, facilitating the detection of small bone lesions. However,^ 99m^Tc-MDP accumulated for the bone repair with limited specificity, whereas ^99m^Tc-3PRGD2 targeted the metastatic tumor directly.

**Figure 4:**
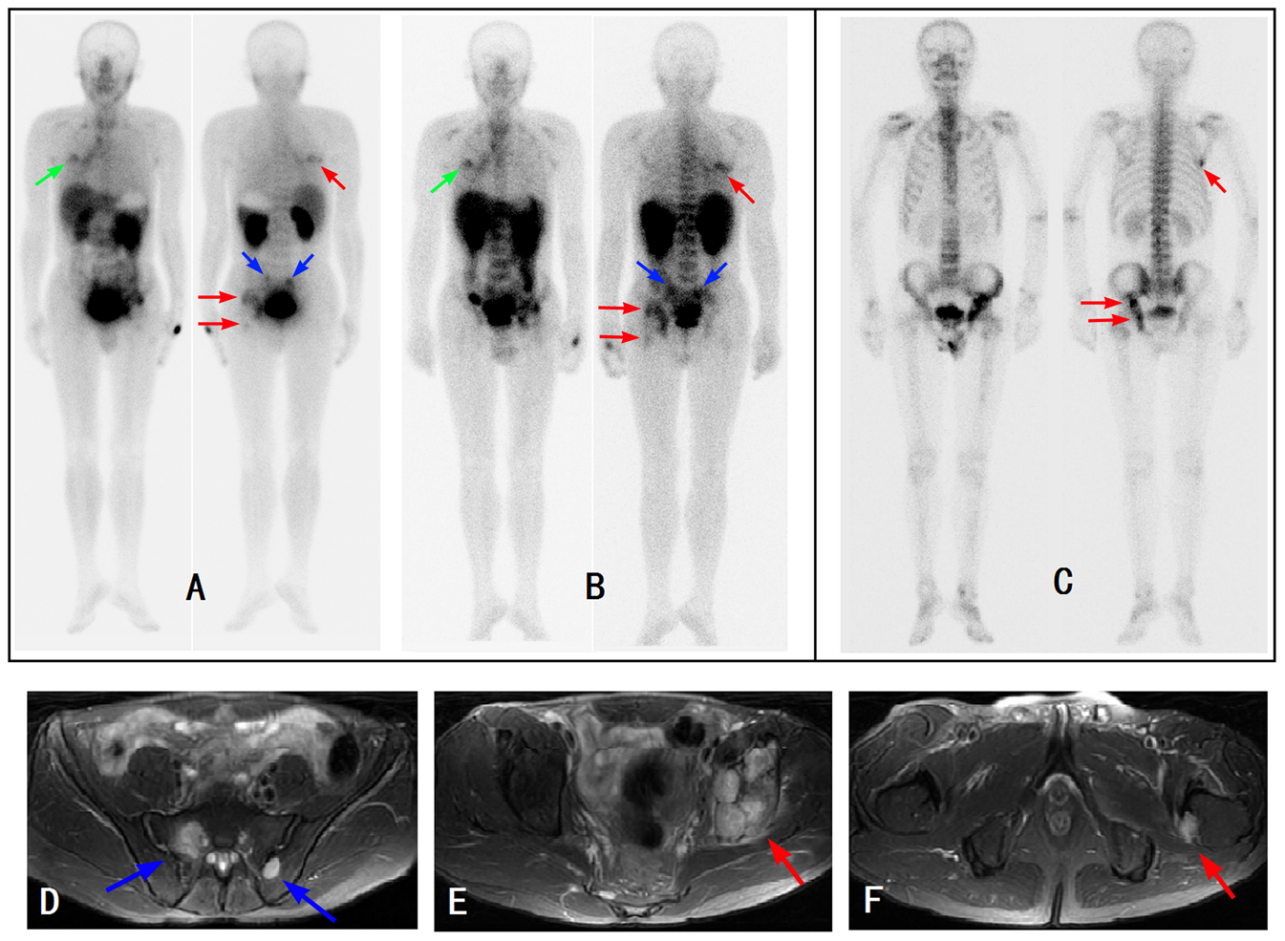
A 48 year-old man with right lung adenocarcinoma (green arrow) and multiple bone metastases (red and blue arrows). ^99m^Tc-3PRGD2 scans showed two more bone metastases (blue arrows) than the ^99m^Tc-MDP bone scan. The lesions were confirmed by MRI (lower row). A: 1-h ^99m^Tc-3PRGD2, B: 4-h ^99m^Tc-3PRGD2, C:^ 99m^Tc-MDP, D–F: MRI.

In the 44 recruited patients, 27 were found with bone lesions. According to the comprehensive assessment, 18 patients were diagnosed with bone metastasis and 9 with benign bone lesion (Fig. 1–4). In the lesion-based analysis of the 27 patients, 112 bone lesions were detected in total, among which 89 were diagnosed as bone metastasis and 23 were benign lesions. The diagnostic efficacy for the assessment of bone metastasis was compared among the 1-h and 4-h ^99m^Tc-3PRGD2 imaging and the ^99m^Tc-MDP whole-body scan (Table 3).

**Table 3:** Comparison of the diagnostic efficacy of ^99m^Tc-3PRGD2 integrin receptor imaging and ^99m^Tc-MDP bone scan for bone metastasis.

	Sensitivity	Specificity	Accuracy	PPV	NPV
**1-h RGD**	**92.1%(82/89)**	**91.3%(21/23)**	**92.0%(103/112)**	**97.6%(82/84)**	**75.0%(21/28)**
**4-h RGD**	**75.3%(67/89)**	**73.9%(17/23)**	**75.0%(84/112)**	**91.8%(67/73)**	**43.6%(17/39)**
**MDP**	**87.6%(78/89)**	**60.9%(14/23)**	**82.1%(92/112)**	**89.7%(78/87)**	**56.0%(14/25)**
a	0.989	5.855	4.793	4.503	2.126
X^2^ b	4.501	0.890	1.697	0.211	0.939
c	9.269	1.362	11.687	1.678	6.550
a	0.320	**0.016**	**0.029**	**0.034**	0.145
*P* b	0.034	0.345	0.193	0.646	0.332
c	**0.002**	0.243	**0.001**	0.195	**0.010**

a:1-h RGD vs. MDP.

b:4-h RGD vs. MDP.

c:1-h vs. 4-h RGD.

PPV: positive predictive value; NPV: negative predictive value; RGD: ^99m^Tc-3PRGD2 imaging; MDP: ^99m^Tc-MDP bone scan.

### Comparison between the 1-h and 4-h ^99m^Tc-3PRGD2 imaging in evaluation of bone metastasis

The 4-h ^99m^Tc-3PRGD2 images showed more prominent uptake in the bone marrow and bowels than the 1-h images, which caused difficulties in identifying bone lesions, especially in the vertebral and pelvic regions (Fig. 3 and 4). According to the lesion-based analysis, the 1-h ^99m^Tc-3PRGD2 imaging revealed more bone lesions than the 4-h imaging. Both false positive and false negative lesions were more common in the 4-h imaging than in the 1-h imaging. On the 1-h images, there were 2 false positive lesions located in thoracic vertebra (n = 2); whereas on the 4-h images, except for the above-mentioned 2 false positive lesions, there were 4 more false positive lesions in the ribs. Seven false negative lesions occurred in the ribs (n = 5), lumbar vertebra (n = 1), and ilium (n = 1) on the 1-h images; whereas on the 4-h images there were totally 22 false negative lesions, including in the ribs (n = 12), lumbar vertebra (n = 6), ilium (n = 2), and other sites (n = 2).

The sensitivity, accuracy and NPV of the 1-h imaging were 92.1%, 92.0% and 75.0% respectively. These values remarkably decreased to 75.3%, 75.0% and 43.6% on the 4-h imaging (*P*<0.05) (Table 3). The specificity and PPV had no significant difference between the 1-h imaging and the 4-h imaging.

### Comparison between the 1-h/4-h ^99m^Tc-3PRGD2 imaging and the ^99m^Tc-MDP scan

The sensitivity of 1-h 99mTc-3PRGD2 imaging was 92.1% for detection of bone metastasis, and there was no statistical difference between the 1-h 99mTc-3PRGD2 imaging and 99mTc-MDP bone scan (P>0.05). Although ^99m^Tc-3PRGD2 imaging failed to spot some small lesions as found on ^99m^Tc-MDP scintigraphy (Fig. 3), it also revealed some lesions undetectable on ^99m^Tc-MDP scintigraphy (Fig. 4). Moreover, the specificity, accuracy, and PPV were 91.3%, 92.0%, and 97.6%, respectively for the 1-h ^99m^Tc-3PRGD2 imaging, significantly higher than those for the ^99m^Tc-MDP bone scan (*P*<0.05). However, the 4-h ^99m^Tc-3PRGD2 imaging showed significantly lower sensitivity (75.3%) than the ^99m^Tc-MDP bone scan (*P*<0.05), whereas no significant difference was found between the two methods in the specificity, accuracy, PPV, and NPV (Table 3).

## Discussion

Integrin α_v_β_3_, an important receptor in the integrin family, is highly relevant to neoplastic angiogenesis. Integrin α_v_β_3_ is highly expressed in neovascular endothelium and many kinds of tumor cells; in contrast, it is rarely found in normal tissues and quiescent endothelium. This characteristic makes it an important target in the diagnosis and treatment of malignancies [9]–[11].^ 99m^Tc-3PRGD2, a novel radiolabeled RGD-based peptide targeting integrin α_v_β_3_ receptor, has recently been translated into clinical application [28]. Compared to the previous RGD-based tracers such as^ 18^F-Galacto-RGD, ^18^F-AH111585, and ^99m^Tc-NC100692, the RGD dimmer structure of^ 99m^Tc-3PRGD2 has significantly enhanced binding affinity to integrin α_v_β_3_, resulting in higher and prominent accumulation in tumors [21]–[27]. Moreover, ^99m^Tc-3PRGD2 is a SPECT tracer that is more cost-effective and can be more widely applied in clinical practice. A recent multi-center study has revealed the importance of ^99m^Tc-3PRGD2 in diagnosis of lung cancer [28]. As a further interest, this study compared ^99m^Tc-3PRGD2 imaging with ^99m^Tc-MDP bone scan in diagnosis of bone metastasis. To the best of our knowledge, it is the first integrin receptor imaging study focused on the evaluation of bone metastasis.

^99m^Tc-MDP whole body scan has been widely used in the detection of bone metastasis with high sensitivity but poor specificity. ^99m^Tc-3PRGD2 can specifically bind to integrin α_v_β_3_ receptor in tumor angiogenesis and various types of tumor cells as well [26], [27], offering a promising radio-labeled tracer that can compensate the insufficiency of ^99m^Tc-MDP bone scan in the diagnosis of bone metastasis. This study showed that the 1-h ^99m^Tc-3PRGD2 imaging had higher specificity, accuracy and PPV than ^99m^Tc-MDP bone scan in diagnosing bone metastasis of lung cancer. However, integrin avb3 is also expressed on osteoclasts and osteoblasts. This may associate with the false positive and false negative conditions in the patients, although much less than those in the ^99m^Tc-MDP bone scans. In this study, false positive lesions mainly occurred in thoracic vertebra and were later diagnosed as degenerative changes via comprehensive analysis of CT, MRI, and follow-ups. As known already, some kinds of inflammation can also have neo-vasculature [29], [30] and may cause ^99m^Tc-3PRGD2 accumulation. The false negative lesions occurred most frequently in ribs and lumbar vertebra. The main reason was the moderate to intense ^99m^Tc-3PRGD2 accumulation in the liver, spleen, bowel, and kidneys. The physiological distribution might make the mild-uptake lesions undetectable on the planar images. However, some of the lesions could be found on the SPECT images. On the other hand, ^99m^Tc-3PRGD2 is a receptor tracer targeting the tumor cells directly. Therefore, ^99m^Tc-3PRGD2 imaging may detect some bone metastases undetectable via the ^99m^Tc-MDP bone scan when no obvious osteogenesis or osteolytic changes exist (Fig. 4).

The sensitivity, accuracy, and NPV were remarkably higher on the 1-h ^99m^Tc-3PRGD2 imaging when compared to those on the 4-h imaging. There were several probable reasons. First, the blood pool background of the 1-h images was low enough. The elimination of ^99m^Tc-3PRGD2 in blood pool was rather fast. Jia B. et al discovered that the ^99m^Tc-3PRGD2 in blood circulation became very low even at 15 minutes [27]. Second, the uptake of the bone marrow and the bowel was higher at 4 h than those at 1 h. The detection of bone lesions was thus more difficult when imaged at 4 h, especially in the lumbar vertebra and pelvic regions. Third, the ^99m^Tc-3PRGD2 uptake in the bone lesions was similar or even relatively decreased at 4 h in this group of patients. Therefore, the 1-h ^99m^Tc-3PRGD2 imaging was usually enough for the detection of bone metastasis, whereas performing another imaging at 4 h was time-consuming and inconvenient with few aids to the diagnosis.

There are some limitations of this study. First, it is a challenge to diagnose bone metastasis without pathological confirmation of each lesion. In this study, we employed the comprehensive analysis of all available data as the gold standard; whereas the readers assessed the lesions blind to the other data. Second, the data came from 4 different centers with different SPECT system, resulting in unavoidable variation in images. However, this study was designed prospectively in a way that all SPECT systems met the quality control and the studies strictly followed the same protocol. All the images passed the quality control before a centralized reading. The third limitation of this study is the relatively small number of patients. Further studies involving more patients are suggested, as the current results preliminarily indicate the value of ^99m^Tc-3PRGD2 imaging as a promising tool to compensate the limited specificity of ^99m^Tc-MDP bone scan. Based on the findings in this study and as an interest in further clarifying the value of ^99m^Tc-3PRGD2 imaging, ^99m^Tc-3PRGD2 imaging may be employed for early assessment of therapeutic response of bone metastasis to compensate the limitation of the conventional imaging methods, including ^99m^Tc-MDP bone scan, CT, and MRI.

In conclusion, this multi-center study indicates that the 1-h ^99m^Tc-3PRGD2 imaging has higher specificity, accuracy, and PPV than the ^99m^Tc-MDP whole body scan in diagnosis of bone metastasis, meriting further studies with more patients. Moreover, the 1-h ^99m^Tc-3PRGD2 imaging is better than the 4-h imaging and is usually enough for the detection of bone metastasis in patients with lung cancer.

## Supporting Information

Checklist S1 **CONSORT checklist for the trial.**
(DOC)

Protocol S1 **Protocol for the trial.**
(PDF)
