# Supplementary material for: Comparison of 99mTc-3PRGD2 Integrin Receptor Imaging with 99mTc-MDP Bone Scan in Diagnosis of Bone Metastasis in Patients with Lung Cancer: A Multicenter Study
Source: PLoS One. 2014 Oct 22;9(10):e111221. doi: 10.1371/journal.pone.0111221 (PMC4206469; doi:10.1371/journal.pone.0111221)
Supplement: Checklist S1 — CONSORT checklist for the trial. (DOC) [file pone.0111221.s001.doc]

**
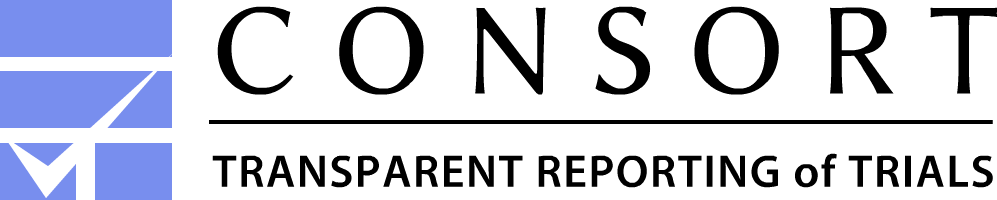
**

**CONSORT 2010 Flow Diagram**

**Allocation**

**Analysis**

**Follow-Up**

**Enrollment**

YES ( )

NO ( √ )

Assessed for eligibility (n= 44 )

Excluded (n= 17)

♦  Not meeting inclusion criteria (n= 17)

♦  Declined to participate (n= 0 )

♦  Other reasons (n= 0 )

Analysed (n= )
♦ Excluded from analysis (give reasons) (n= )

Lost to follow-up (give reasons) (n= )

Discontinued intervention (give reasons) (n= )

Allocated to intervention (n= )

♦ Received allocated intervention (n= )

♦ Did not receive allocated intervention (give reasons) (n= )

Lost to follow-up (give reasons) (n= 0 )

Discontinued intervention (give reasons) (n= 0 )

Allocated to intervention (n= 27 )

♦ Received allocated intervention (n= 27)

♦ Did not receive allocated intervention (give reasons) (n= 0 )

Analysed (n= 27 )
♦ Excluded from analysis (give reasons) (n= 0 )

Is it randomized?
