# Supplementary material for: Comparison of 99mTc-3PRGD2 Integrin Receptor Imaging with 99mTc-MDP Bone Scan in Diagnosis of Bone Metastasis in Patients with Lung Cancer: A Multicenter Study
Source: PLoS One. 2014 Oct 22;9(10):e111221. doi: 10.1371/journal.pone.0111221 (PMC4206469; doi:10.1371/journal.pone.0111221)
Supplement: Protocol S1 — Protocol for the trial. (PDF) [file pone.0111221.s002.pdf]

^{99m}Tc-3PRGD2 SPECT in evaluation of bone metastasis in lung cancer patients: compared with ^{99m}Tc-MDP bone scan

Aims:

This study was a part of a multi-center study designed to evaluate the diagnostic accuracy of ^{99m}Tc-3PRGD2 imaging in lung cancer patients. The aim of this sub-topic was to compare ^{99m}Tc-3PRGD2 imaging with ^{99m}Tc-MDP bone scan in evaluation of bone metastasis in lung cancer patients.

The medical centers and facilities:

Centers	SPECT system
The 1st Affiliated Hospital of Fujian Medical University, Fuzhou	GE Infinia Hawkeye
Beijing Tongren Hospital, Beijing	GE Infinia Hawkeye4
The 1st Affiliated Hospital of Nanjing Medical University, Nanjing	Siemens Symbia T6
Peking Union Medical College Hospital, Beijing (the organizer center)	Philips Precedence 16

Estimated Enrollment: 40

Study Start Date: February 2011

Estimated Study Completion Date: March 2014

Introduction:

Integrin is mainly involved in the cell-cell and cell-matrix interactions. Integrin $\alpha_V\beta_3$, an important member of the integrin family, is highly relevant to neoplastic angiogenesis, invasion, and metastasis. It is not expressed at all or expressed at a very low level on the quiescent endothelium and other normal tissues. Therefore, the integrin $\alpha_V\beta_3$ receptor may be a promising target in the diagnosis, evaluation, and treatment of malignancies, serving as a tumor-specific agent for single photon emission computed tomography (SPECT).

The tri-peptide sequence of arginine-glycine-aspartic acid (RGD), especially in

cyclic form, has shown high affinity in binding with the receptors of integrin $\alpha_V\beta_3$. ^{99m}Tc-3PRGD2 could be easily prepared and exhibited excellent in vivo behaviors in animal models. No adverse reactions are observed in animal models to date.

For further interest in clinical translation of ^{99m}Tc-3PRGD2, this study was designed to investigate the value of ^{99m}Tc-3PRGD2 in assessment of bone metastasis in lung cancer patients, which was compared with ^{99m}Tc-MDP.

Methods

A single dose of 11.1 MBq/kg body weight ^{99m}Tc-3PRGD2 will be intravenously injected into the patients, and then whole body planar scan (10 cm/min) and chest SPECT imaging (zoom ×1, 30 sec/frame/6°) will be performed at dual time-points of 1 h and 4 h. ^{99m}Tc-MDP whole body imaging will be done for comparison within 1 week before or after ^{99m}Tc-3PRGD2 imaging. Consensus reading of ^{99m}Tc-3PRGD2 and ^{99m}Tc-MDP images will be performed on the same workstation by three experienced nuclear medicine physicians blind to the history and pathological diagnosis. The sensitivity, specificity, accuracy, positive predictive value, and negative predictive value will be employed to determine the efficacy of ^{99m}Tc-3PRGD2 imaging for the diagnosis of bone metastases.

Eligibility

Inclusion Criteria:

1. Males and females, ≥30 years old
2. Patients suspected with primary lung cancer with thoracic CT
3. The lung cancer will be histologically confirmed or results of histology will be available.
4. ^{99m}Tc-MDP whole body scan will be performed within 1 week before or after ^{99m}Tc-3PRGD2 imaging.
5. The bone lesion will be confirmed from at least 2 imaging modalities (SPECT/CT/MRI/^{18}F-FDG PET-CT) and 6-month follow-up.

Exclusion Criteria:

1. Females planning to bear a child recently or with childbearing potential
2. Known severe allergy or hypersensitivity to IV radiographic contrast
3. Inability to complete the needed examination due to severe claustrophobia, radiation phobia, etc.
4. Concurrent severe and/or uncontrolled and/or unstable other medical disease

that, in the opinion of the investigator, may significantly interfere with compliance

Flow Chart

RESEARCH PROCESS	V1	V2	V3	V4 (Surgery)	V5 (Follow-up)
Window period		Within two weeks before the surgery	Within one week before/after the ^{99m}Tc-3PRGD2 imaging		Every 3-6 months
Inclusion / Exclusion criteria	X				
Signed the Informed Consent	X				
Medical history / Concomitant diseases	X				
CT, MRI, PET/CT	X				
Tumor markers and other laboratory tests	X				
^{99m}Tc-3PRGD2 SPECT		X			
^{99m}Tc-MDP whole body scan			X		
Adverse events		X			
Surgical records and pathology results				X	
Preserve specimens				X	
Subsequent treatment and follow-up					X

Contacts and Locations

1. Zhaohui Zhu
Department of Nuclear Medicine, Peking Union Medical College Hospital, Chinese Academy of Medical Science & Peking Union Medical College, Beijing, 100730
Phone: 86-10-69154196, E-mail: zhuzhh@pumch.cn

2. Weibing Miao
Department of Nuclear Medicine, the 1st Affiliated Hospital of Fujian Medical University
No 20 Chazhong Road, Taijiang District, Fuzhou, Fujian, 350005
Phone: 86-591-87981618, E-mail: miaoweibing@126.com
